# Genome-wide identification of alternative splicing and splicing regulated in immune infiltration in osteosarcoma patients

**DOI:** 10.3389/fgene.2023.1051192

**Published:** 2023-04-17

**Authors:** Zhibing Dai, Yachao Sun, Maierdanjiang Maihemuti, Renbing Jiang

**Affiliations:** Department of Bone and Soft Tissue, Affiliated Tumor Hospital of Xinjiang Medical University, Urumqi, China

**Keywords:** osteosarcoma (OS), alternative splicing (AS), regulated alternative splicing (RAS), RNA-Seq, immune cells, RNA-binding proteins (RBPs)

## Abstract

**Background:** Osteosarcoma typically occurs in adolescents, and the survival rate of patients with metastatic and recurrent osteosarcoma remains low. Abnormal regulation of alternative splicing is associated with the development of osteosarcoma. However, there is no genome-wide analysis of the function and regulatory mechanisms of aberrant alternative splicing associated with osteosarcoma.

**Methods:** Published transcriptome data on osteosarcoma (GSE126209) derived from osteosarcoma patient tissue were downloaded. Gene expression profiling by high-throughput sequencing was performed on 9 normal samples and 10 tumor samples for genome-wide identification of osteosarcoma-related alternative splicing events. The potential function of osteosarcoma-associated alternative splicing events was examined by immune infiltration and correlation analysis. Regulation of aberrantly expressed RNA-binding proteins (RBPs) related to alternative splicing in osteosarcoma was clarified by co-expression analysis.

**Results:** A total of 63 alternative splicing events, which are highly credible and dominant, were identified. GO enrichment analysis indicated that alternative splicing may be closely related to the immune response process. Immune infiltration analysis showed significant changes in the percentages of CD8 T cells, resting memory CD4 T cells, activated memory CD4 T cells, monocytes, resting dendritic cells, and activated mast cells in tumors compared to normal tissues, indicating the involvement of these immune cell types in the occurrence of osteosarcoma. Moreover, the analysis identified alternative splicing events that were co-altered with resting memory CD4 T cells, resting dendritic cells, and activated mast cells, events that may be associated with regulation of the osteosarcoma immune microenvironment. In addition, a co-regulatory network (RBP-RAS-immune) of osteosarcoma-associated RBPs with aberrant alternative splicing and altered immune cells was established. These RBPs include NOP58, FAM120C, DYNC1H1, TRAP1, and LMNA, which may serve as molecular targets for osteosarcoma immune regulation.

**Conclusion:** These findings allow us to further understand the causes of osteosarcoma development and provide a new research direction for osteosarcoma immunotherapy or targeted therapy.

## Introduction

Osteosarcoma (OS) is a malignant bone tumor frequently occurring in children and adolescents (Durfee et al., 2016). Treatment of OS usually involves chemotherapy and surgical resection. Chemotherapy improves the survival rate to some extent. The 5-year survival rate of patients with osteosarcoma is about 70%, while the 5-year overall survival rate of patients with metastasis or recurrence is significantly reduced, even less than 20% (Harrison et al., 2018). Overall, the prognosis of patients with OS has not changed significantly for more than 30 years (Smrke et al., 2021). Advanced, recurrent, or metastatic OS is still difficult to cure. Due to the biological complexity of OS, traditional techniques are not optimal, resulting in no new improvement in treatment in recent years. In view of this, new methods for OS treatment need to be developed.

Alternative splicing (AS) is closely related to the functional complexity of eukaryotes (Pan et al., 2008). AS is an important process that is involved in the production of proteome diversity in eukaryotic cells. Abnormal splicing is related to the occurrence and development of cancer (Nilsen and Graveley, 2010). Cancer-related AS events can be used as prognostic factors for disease and as therapeutic targets (Zhao et al., 2022). For example, Rothzerg found that AS events in the *LEPROT* gene may be an important factor in the development of OS (Rothzerg et al., 2020). Nevertheless, there are no studies analyzing the function of OS-associated aberrant AS on a genome-wide scale.

RNA-binding proteins (RBPs) are a general term for a class of proteins that are associated with the regulation of RNA metabolism and RNA binding. RBPs recognize special RNA-binding domains to interact with RNA and participate in various post-transcriptional regulatory processes, such as RNA splicing, transport, polyadenylation, intracellular localization, translation, and degradation (Glisovic et al., 2008). Regulation of RBPs is related to various biological processes in cells (Cainap et al., 2015). Previous studies have shown that RBPs play an important role in the development of many diseases, including cancer (Cabibbo et al., 2010), and abnormal expression of RBPs is closely related to the prognosis of tumor patients (Dreyfuss et al., 2002; Huang et al., 2014). The RNA-binding protein MSI1 is more highly expressed in OS tissues than in adjacent tissues. The knockdown of MSI1 in OS cells inhibits their proliferation and tumorigenesis (Niu et al., 2017). The expression of PTBP1 in chemotherapy-resistant OS tissues is significantly higher than that in chemotherapy-sensitive OS tissues (Cheng et al., 2020). AS is a very important process in post-transcriptional regulation of RBPs, and AS makes gene expression more complex, increases transcription efficiency, promotes protein diversity, and plays a significant role in cell differentiation and disease (Venables, 2004). However, the mechanism by which RBPs regulate AS in OS has not yet been elucidated.

We hypothesized that after the occurrence of OS, a large number of RBPs are abnormally regulated, and then, these regulate the AS of gene pre-mRNAs, which may cause different AS events to produce different proteins, playing a regulated role in the development of OS by affecting the immune microenvironment. For this study, we downloaded published transcriptome data for OS (GSE126209) and analyzed the gene expression profile of 9 normal samples and 10 tumor samples by high-throughput sequencing. The regulatory changes in AS in OS and the regulated relationship between AS and changes in immune cells were revealed. Co-expression analysis was also carried out, and the RBP-RAS regulated network and immune cells related to OS were established.

## Materials and methods

### Retrieval and process of public data

Public sequence data files were downloaded from Sequence Read Archive (SRA). The SRA Run files were converted to FASTQ format with the NCBI SRA Tool fastq-dump. The raw reads were trimmed of low-quality bases using the FASTX-Toolkit (v.0.0.13; http://hannonlab.cshl.edu/fastx_toolkit/). The clean reads were evaluated using FastQC (http://www.bioinformatics.babraham.ac.uk/projects/fastqc).

### Read alignment and differentially expressed gene analysis

Clean reads were aligned to the human genome by HISAT2 (Kim et al., 2015), and the human genome version is GRCh38. Uniquely mapped reads were ultimately used to calculate read number and fragments per kilobase of exons per million fragments mapped (FPKM) for each gene. The expression levels of genes were evaluated using FPKM. When we carried out gene differential expression analysis, we had chosen DESeq2_ 1.30.1 software (Love et al., 2014). DEseq2 will model the original reads and use the scale factor to explain the difference of the library depth. Then, DESeq2 estimates the gene dispersion and reduces these estimates to generate more accurate dispersion estimates so as to model the read count. Finally, the model of negative binomial distribution is fitted by DESeq2, and the hypothesis is tested by the Wald test or likelihood ratio test. DESeq2 can be used to analyze the differential expression between two or more samples, and the analysis results can be used to determine whether a gene is differentially expressed by the fold change (FC) and false discovery rate (FDR).

**There are two important parameters**
1) FC: Fold change, the absolute ratio of expression change.
2) FDR: False discovery rate.
**The criteria of significant difference expression were as follows**

FC≥2 or≤0.5,FDR≤0.05.

### Identification of differentially expressed RBPs in the groups

DESeq2_ 1.30.1 software, which is specifically used to analyze the differential expression of genes, was used to screen the raw count data for differentially expressed genes (DEGs). The results were analyzed based on the fold change (FC ≥ 2 or ≤0.5) and false discovery rate (FDR≤0.05) to determine whether a gene was differentially expressed. Then, the expression profile of differentially expressed RBPs was filtered out from all DEGs according to a catalog of 2,141 RNA-binding proteins (RBPs) retrieved from four previous reports (Castello et al., 2012; Gerstberger et al., 2014; Castello et al., 2016; Hentze et al., 2018).

### Alternative splicing analysis

Regulated alternative splicing (RAS) was defined and quantified using the splice-site usage variation analysis (SUVA) pipeline (Cheng et al., 2021). Differential splicing in each group was analyzed. The read proportion of SUVA AS events (pSAR) of each AS event was calculated.

### Co-expression analysis

Co-expression analysis was performed for all differentially expressed RBPs and RASs (pSAR≥50%). The Pearson correlation coefficient between differentially expressed RBPs and RASs was calculated, and RBP-RAS relationship pairs satisfying the absolute value of the correlation coefficient ≥0.8 and *p*-value ≤0.01 were screened. Co-expression analysis was performed for all differentially expressed RBPs and RASs (pSAR ≥50%) and immune cells. The Pearson correlation coefficient between differentially expressed RBPs and RAS and immune cells was calculated, and RBP-RAS-immune cell relationship pairs satisfying the absolute value of the correlation coefficient ≥0.8 and *p*-value ≤0.01 were screened.

### Cell-type quantification

The CIBERSORT algorithm (Newman et al., 2015) was used with default parameters to estimate immune cell fractions using FPKM values of each expressed gene. A total of 22 human immune cell phenotypes were analyzed, including the following: six T-cell types [CD8 T cells, naïve CD4 T cells, memory CD4 resting T cells, memory CD4 activated T cells, T follicular helper cells, and regulatory T cells (Tregs)]; naïve and memory B cells; plasma cells; resting and activated NK cells; monocytes; macrophages M0, M1, and M2; resting and activated dendritic cells; resting and activated mast cells; eosinophils; and neutrophils.

### Functional enrichment analysis

To explore functional categories of DEGs, Gene Ontology (GO) terms and Kyoto Encyclopedia of Genes and Genomes (KEGG) pathways were identified using the KOBAS 2.0 server (Xie et al., 2011).

The hypergeometric test and Benjamini‒Hochberg FDR controlling procedure were used to define the enrichment of each term.

### Other statistical analyses

Principal component analysis (PCA) was performed using the R package factoextra (https://cloud.r-project.org/package=factoextra) to show the clustering of samples with the first two components. After normalizing reads by the TPM (tags per million) of each gene in the samples, in-house script (Sogen) was applied for visualization of next-generation sequence data and genomic annotations. The pheatmap package (https://cran.r-project.org/web/packages/pheatmap/index.html) in R was used to perform the clustering based on Euclidean distances. Student’s t test was used for comparisons between two groups.

## Results

### Identification of osteosarcoma-associated RAS events (osteosarcoma RAS) between tumor samples and adjacent normal tissues

**FIGURE 1:**
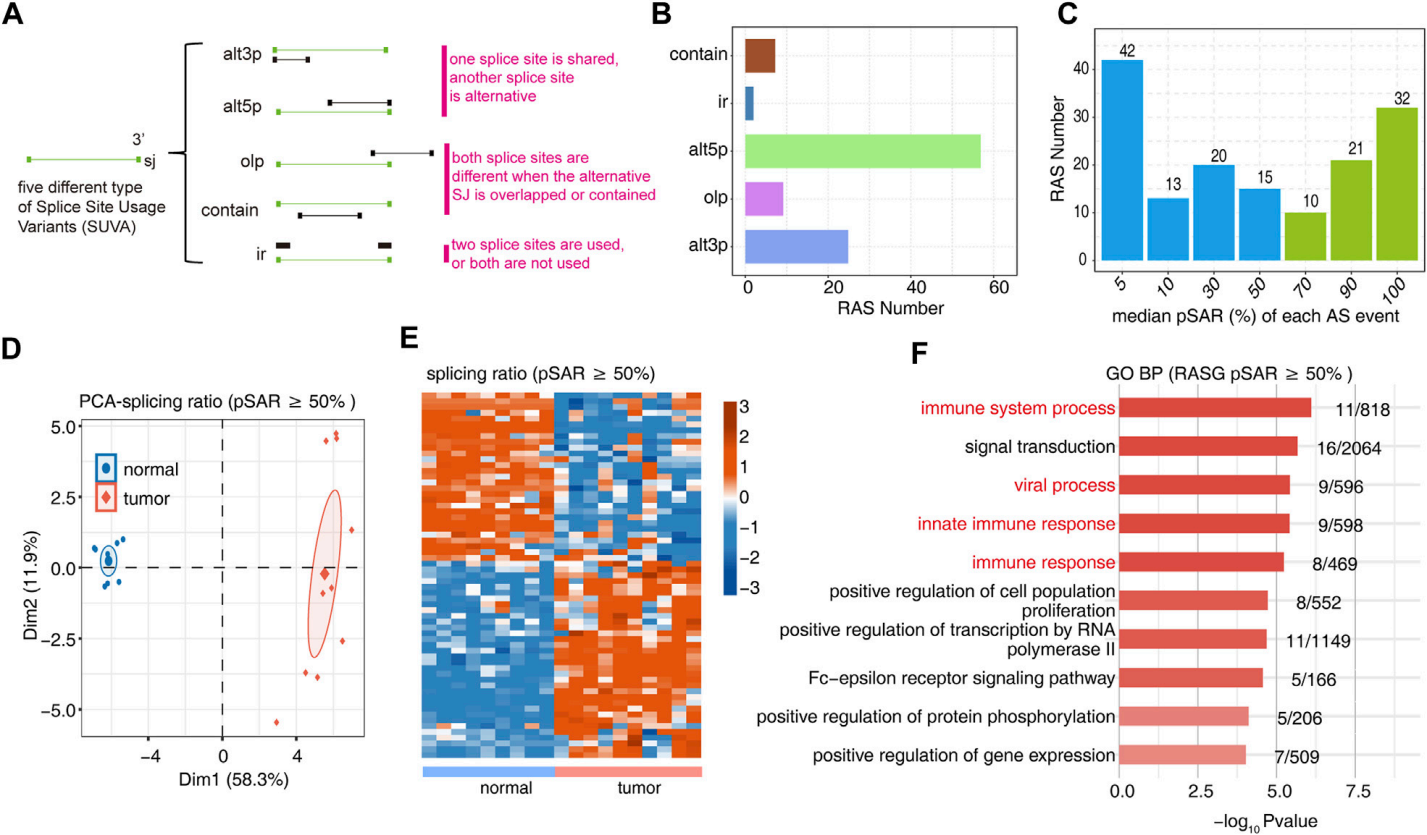
Identification of RAS events between tumor and normal tissues. **(A)** Five different types of AS event models. **(B)** Barplot showing the number of regulatory AS detected by SUVA in each group. **(C)** Barplot showing the RAS number with different pSAR. RAS with pSAR (reads proportion of the SUVA AS event) ≥50% were labeled. **(D)** Principal component analysis (PCA) based on RAS of pSAR ≥50%. The ellipse for each group is the confidence ellipse. **(E)** Heatmap presenting five classes of RAS (pSAR ≥50%). **(F)** The top 10 most enriched GO terms (biological process) were illustrated for RAS (pSAR ≥50%) genes in the tumor vs. normal group. The color scale showing the row-scaled significance (-log10 corrected *p*-value) of the terms.

We used SUVA software (https://doi.org/10.1080/15476286.2021.1940037) to perform the analysis of RNA-Seq data for 9 normal tissues and 10 OS tumor tissues to identify AS events that are significantly different between tumor and normal tissue. Five significantly different regulated alternative splicing events were identified between tumor and normal tissue (Figure 1A, Supplementary Figure S1A). The figure shows the number of RAS detected by SUVA in each group (Figure 1B). A splicing event involves two transcripts, and these two transcripts may only account for a very small part of the entire gene expression. We hope to find more dominant splicing transcripts. The figure shows the number of splicing events that account for different proportions of all reads in the region, and some splicing events account for only a small proportion (pSAR<10%).The figure shows the number of splicing events that account for a different proportion of all reads in the region. We selected RAS (green bar, 63 in total) with pSAR≥50% for downstream analysis in this study (Figure 1C). PCA with the splicing ratio of differential AS events showed that the principal components of the tumor and normal groups could be well separated (Figure 1D). A heatmap displayed the differential splicing event ratio (Figure 1E). Functional pathway analysis was performed on the genes involved in the differential splicing events between the tumor and normal groups, with enrichment mainly in biological pathways such as immune system processes and immune response and cell proliferation (Figure 1F, Supplementary Figure S1B).

### Diversity of immune microenvironment characteristics between tumor and normal samples from osteosarcoma patients

**FIGURE 2:**
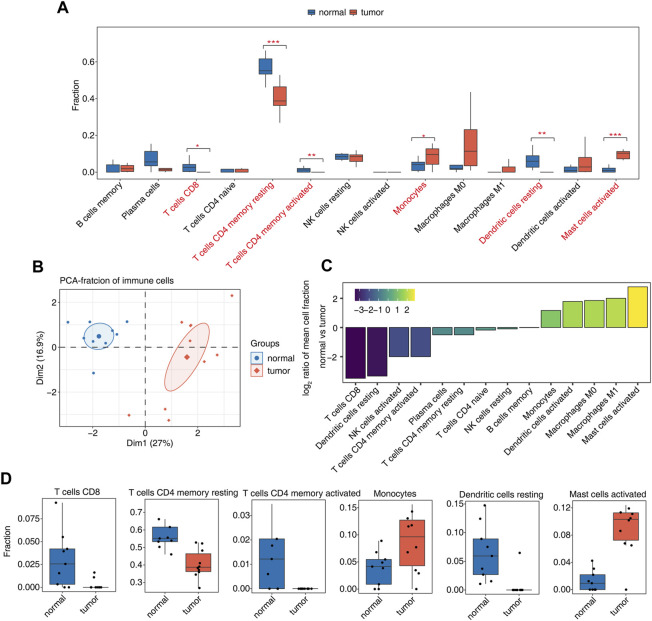
Diversity of immune microenvironment characteristics between tumor and normal samples from osteosarcoma patients. **(A)** Boxplot showing the fraction of each cell type in each group. The significant difference in the immune cell fractions between tumor and normal samples was calculated by the Student’s t test. *, *p*-value ≤0.05; **, *p*-value ≤0.01; ***, *p*-value ≤0.001. **(B)** Principal component analysis (PCA) based on the fractions of different cells of all expressed genes. The ellipse for each group is the confidence ellipse. **(C)** Tumor relative to the normal group rank ordered based on decreasing values of the relative frequency ratio at tumor *versus* normal groups. **(D)** Boxplot showing six significantly immune cells.

Our previous studies have shown that AS may be closely related to the immune response. We further analyzed the proportions of immune cells in tumor and normal samples and the proportions of various types of immune cells. Among them, CD8 T cells, resting memory CD4 T cells, activated memory CD4 T cells, monocytes, resting dendritic cells, and activated mast cells were significantly different (Figure 2A, Supplementary Figure S2A). PCA using the differential ratio of immune cells showed that the immune cells in the tumor and normal groups were well separated (Figure 2B, Supplementary Figure S2B). Another way is to show the difference of each cluster of immune cells in tumor and normal samples, calculate the difference ratio of each cluster, and then take the log2 value; the positive bar represents the increased cluster in OS tumors, and the negative bar represents the decreased cluster. The most significant upregulation was observed for activated mast cells; CD8 T cells and resting dendritic cells were downregulated most significantly (Figure 2C). The difference in the proportions of CD8 T cells, resting memory CD4 T cells, activated memory CD4 T cells, monocytes, resting dendritic cells, and activated mast cells in tumor tissues and normal tissues is shown in Figure 2D. These studies indicate that the function of these immune cell types may be related to the occurrence and development of OS.

### Identification of immune-associated osteosarcoma RAS

**FIGURE 3:**
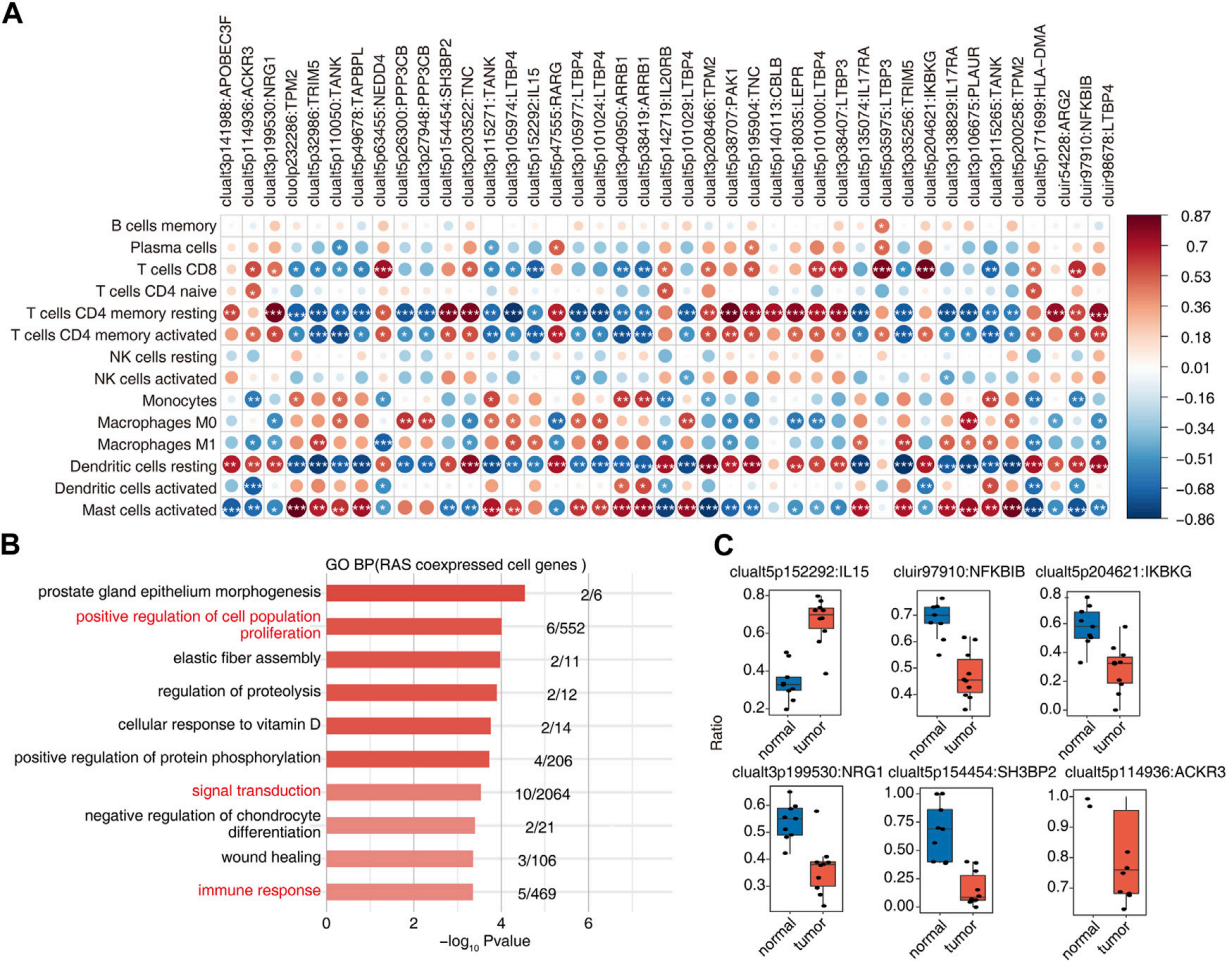
Identification of immune-associated osteosarcoma RAS. **(A)** The dot plot demonstrated the correlations between each immune microenvironment infiltration cell type and each dysregulated RBP regulator. Different colors indicate correlation of the immunocyte–RBP regulator, and significant regulators were labeled with star. *, *p* < =0.05; **, *p* < =0.01; ***, *p* < =0.001. **(B)** The top 10 most enriched GO terms (biological process) were illustrated for RAS coexpressed cell genes in the tumor vs. normal group. The color scale indicates the row-scaled significance (-log10 corrected *p*-value) of the terms. **(C)** Boxplot showing the splicing ratio of clualt3p199530 NRG1, clualt5p154454 SH3BP2, clualt5p114936 ACKR3, clualt5p152292 IL15, cluir97910 NFKBIB, and clualt5p204621 IKBKG. *p < 0.001, **p < 0.001, and ***p < 0.001.

Association analysis of differential AS events and changes in the proportion of immune cells in OS tumor and normal samples yielded 41 RAS (regulatory AS) events that correlated significantly with changes in at least one immune cell (Figure 3A). GO enrichment analysis of AS events related to immune cells showed them to be mainly enriched in biological pathways related to cell proliferation, signal transduction, and immune response (Figure 3B). Ratio differences of important immune-related AS events (clualt3p199530 NRG1, clualt5p154454 SH3BP2, clualt5p114936 ACKR3, clualt5p152292 IL15, cluir97910 NFKBIB, and clualt5p204621 IKBKG) between tumor and normal tissues are presented (Figure 3C, Supplementary Figures S3A–D).

### Identification of differentially expressed RBPs co-disturbed with immune-related osteosarcoma RAS

**FIGURE 4:**
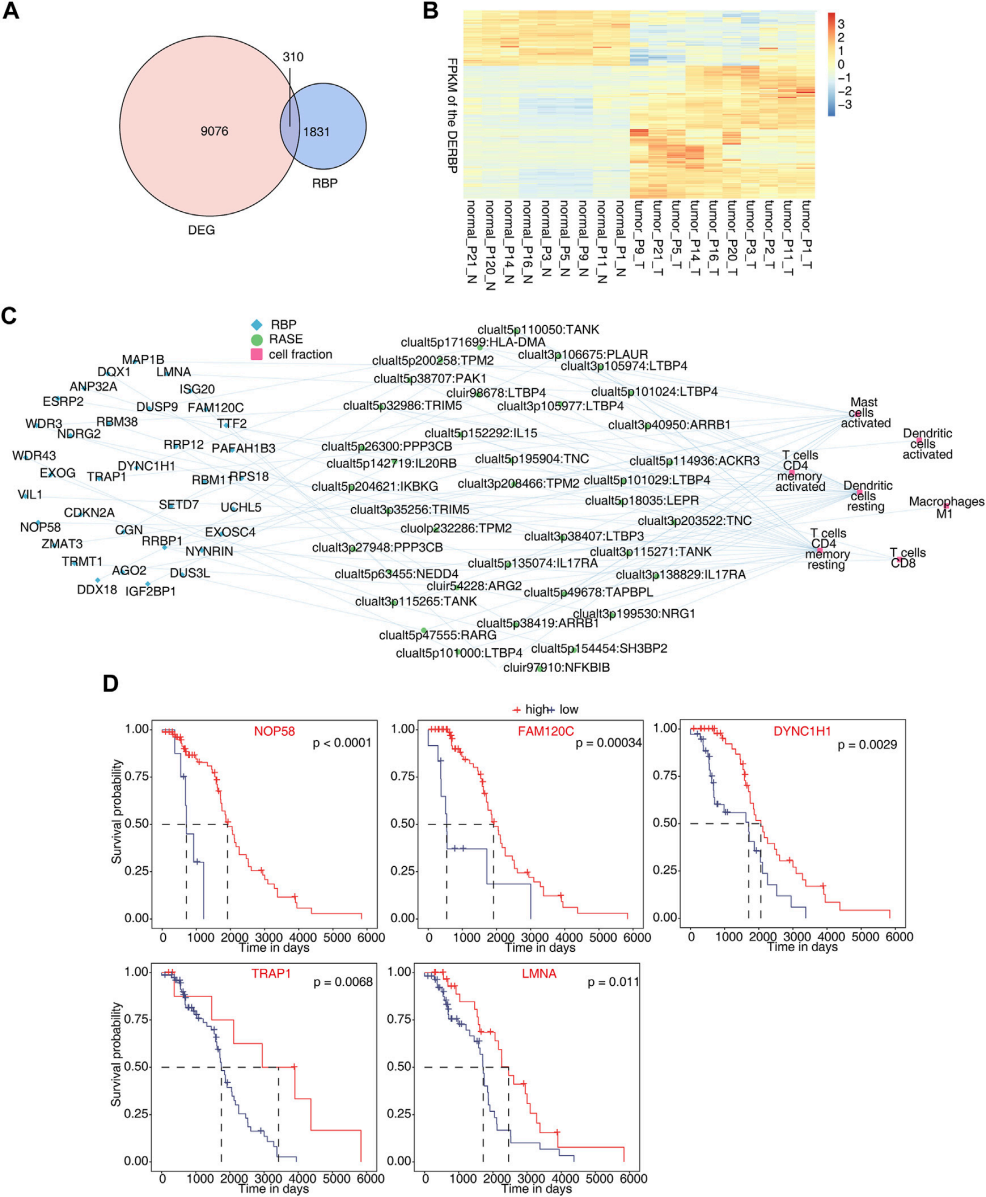
Identification of differentially expressed RBPs co-disturbed with immune-related osteosarcoma-RAS. **(A)** Venn diagram showing the overlap gene number of RBPs and DEGs. **(B)** Hierarchical clustering heatmap showing the FPKM of the DERBPs. **(C)** Network showing the immune cells regulated by DERBP and RASEs. **(D)** Survival curves regulated by NOP58, FAM120C, DYNC1H1, TRAP1, and LMNA.

In total, 310 differentially expressed RBP genes were obtained from the intersection of differentially expressed genes and known RBP genes (Castello et al., 2012; Gerstberger et al., 2014; Castello et al., 2016; Hentze et al., 2018) (Figure 4A). Heatmap analysis and the display of different RBPs showed that about two-thirds of RBPs were upregulated in tumor tissues. This indicates that these RBP genes are more activated in osteosarcoma (Figure 4B). By analyzing RBPs related to immune cells and establishing the regulatory network of RBP-RAS and immune cells, a large number of RBPs were found to be changed in immune cells (Figure 4C). We downloaded the TARGET database (https://ocg.cancer.gov/programs/target/data-matrix) expression data and prognosis information in OS. The RBPs (NOP58, FAM120C, DYNC1H1, TRAP1, and LMNA) related to prognosis are displayed in Figure 4D and Supplementary Figures S4A, B.

## Discussion

We performed an overall AS analysis on the RNA-Seq data in OS, and 63 highly credible and dominant AS events were identified. These AS events are closely related to the immune response process. These RAS events are mainly enriched in biological pathways such as immune system processes, immune responses, and cell proliferation. Then, we conducted immune infiltration analysis based on enrichment of differential AS genes in immune-related pathways. We analyzed the immune cells of OS tumor and normal samples to obtain different proportions of immune cells. It was found that the proportions of CD8 T cells, resting memory CD4 T cells, activated memory CD4 T cells, monocytes, resting dendritic cells, and activated mast cells changed significantly. In addition, we used bar plots to further investigate the proportion of cell types with significant differences between tumor and normal samples among total immune cells. Our study shows that these immune cells play a significant role in the development of OS. Previous studies have shown that the extent of intratumoral CD8 T-cell infiltration is strongly associated with better OS outcomes (Fritzsching et al., 2015). The activation of memory CD4 T-cell-associated genes CST7, CD5L, hsa-miR-23b-3p, and hsa-miR-23a-3p may correlate with the prognosis of hepatocellular carcinoma (Yan et al., 2022). Patrol monocyte levels may play a key role in whether OS patients develop metastasis (Hanna et al., 2015; Chen and Zhao, 2020).

Some studies have shown that AS plays a significant role in the immune microenvironment (Kim et al., 2017; Tan et al., 2017). We first analyzed the correlation between changes in AS and the proportion of immune cell types and found 41 RAS events to be significantly associated with changes in at least one immune cell type. The study found that these alternative splicing events were significant covariants of activated mast cells, resting dendritic cells, and resting CD4 T cells. This finding indicates that these AS events may be related to the regulation of the OS immune microenvironment.

In addition, we identified 310 differentially expressed RBPs in OS tumor and normal tissue samples. Heatmap analysis of these differentially expressed RBPs revealed that RBP expression was activated during OS development. By establishing a regulatory network of RBP, RASs, and immune cells, we found that abnormal expression of RBPs may regulate AS of related genes in the immune pathway, thereby affecting changes in AS of downstream genes. We downloaded the expression data and prognostic information for OS from the TARGET database (https://ocg.cancer.gov/programs/target/data-matrix) and found that NOP58, FAM120C, DYNC1H1, TRAP1, and LMNA may be associated with OS prognosis.

Our research also has some limitations. First, the number of samples is small. However, this study verified differential RBP and AS events in a clinical setting, and the biological significance of OS needs to be confirmed through functional experiments.

## Conclusion

We are the first to analyze genome-wide AS in OS. Sixty-three highly credible and dominant AS events were identified that were mainly enriched in immune response-related biological pathways. AS events were associated with the regulation of the OS immune microenvironment. There is a co-alteration regulated network involved in AS, immune cells, and RBPs. NOP58, FAM120C, DYNC1H1, TRAP1, and LMNA may serve as molecular targets for OS immune regulation; this may contribute to targeted therapy in clinical practice.

## Data Availability

Publicly available datasets were analyzed in this study. These data can be found at: http://www.ncbi.nlm.nih.gov/geo/GSE126209.
