# Supplementary figures and images for: Genome-wide identification of alternative splicing and splicing regulated in immune infiltration in osteosarcoma patients
Source: Front Genet. 2023 Apr 17;14:1051192. doi: 10.3389/fgene.2023.1051192 (PMC10149916; doi:10.3389/fgene.2023.1051192)

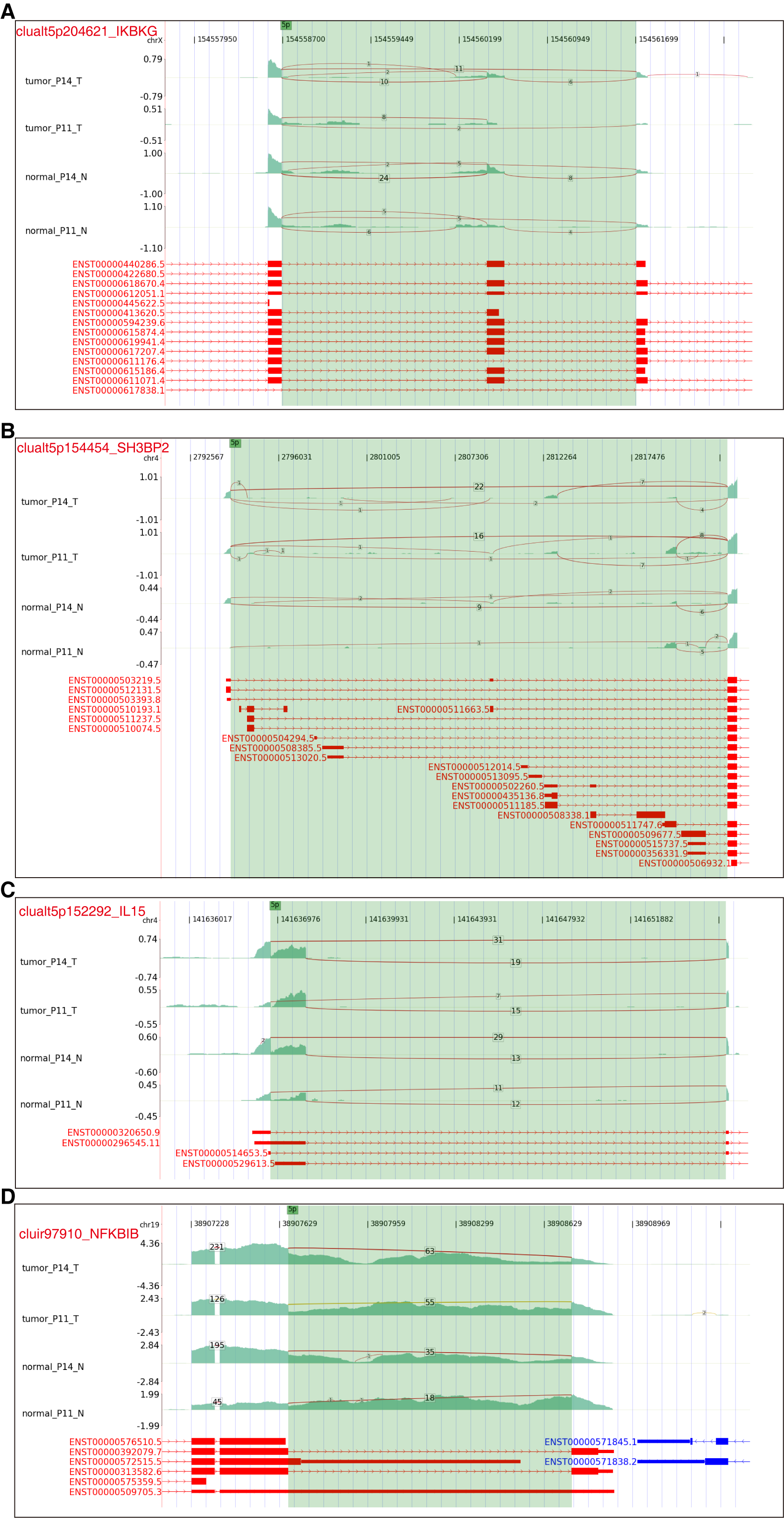

Supplement: Supplementary file 1 [file Image3.TIF]

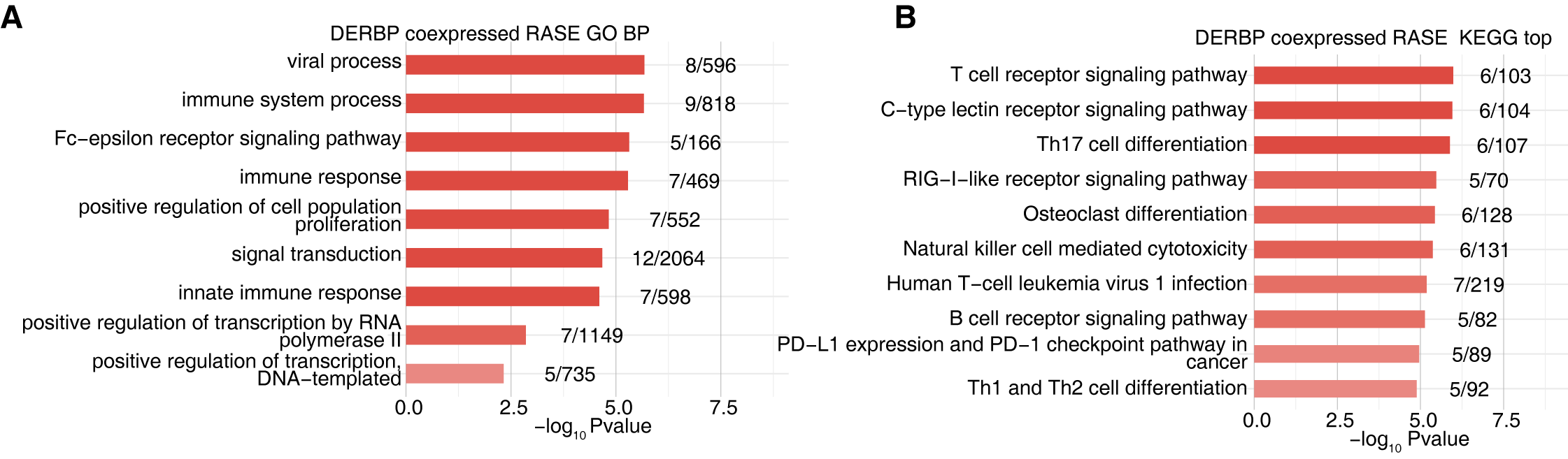

Supplement: Supplementary file 2 [file Image4.TIF]

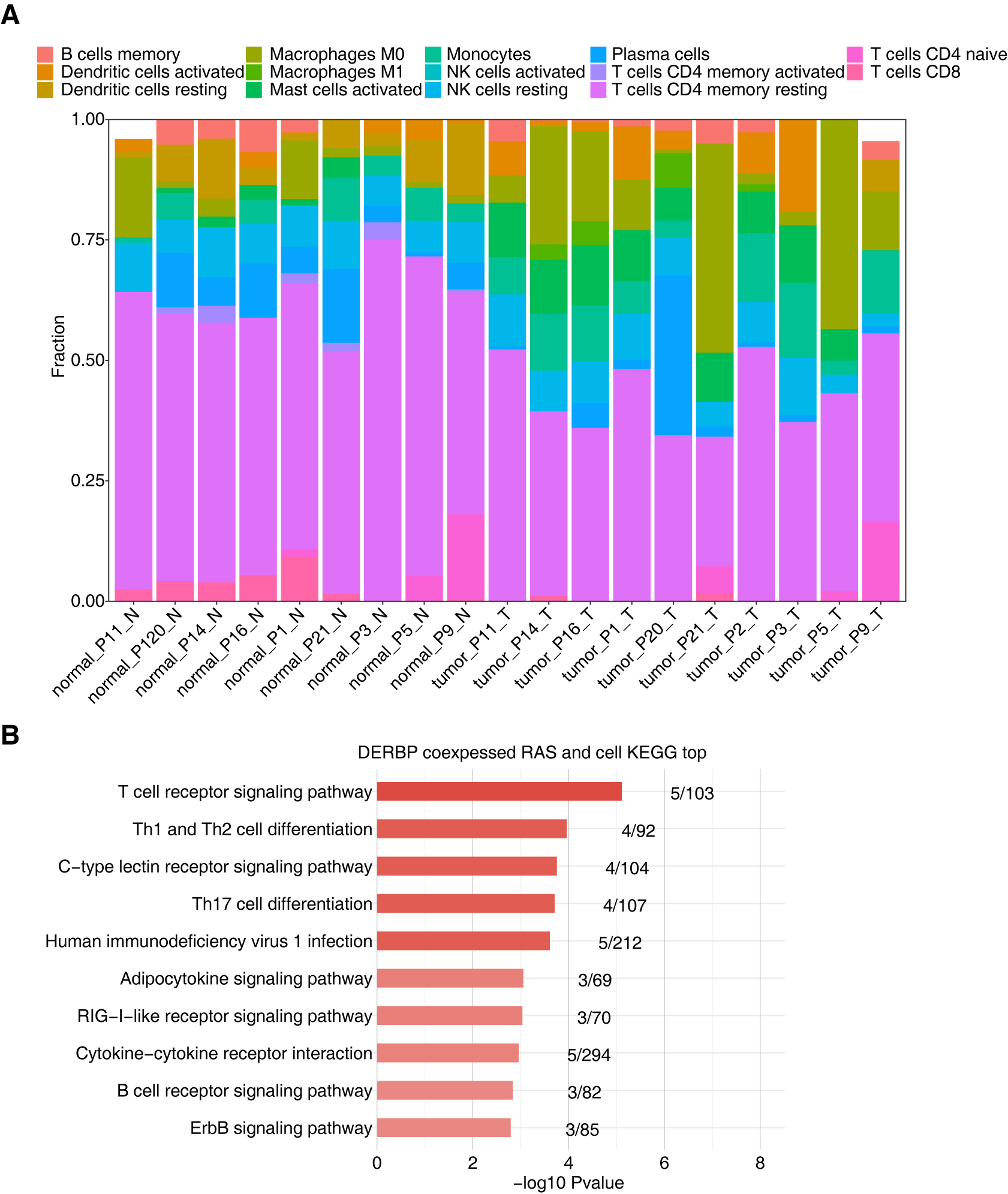

Supplement: Supplementary file 3 [file Image2.TIF]

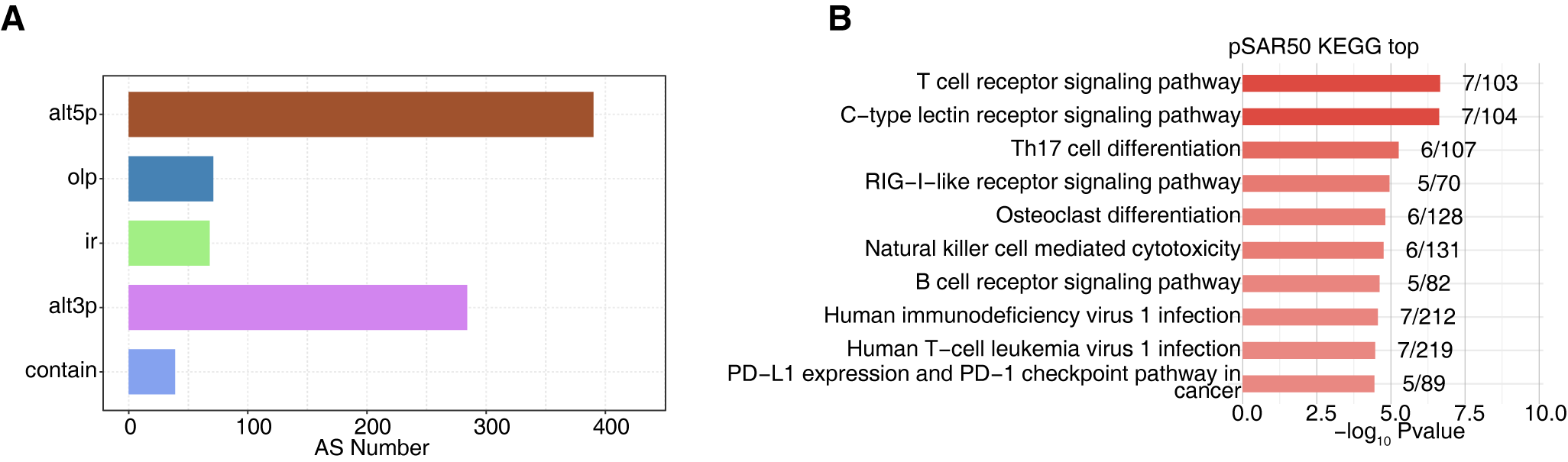

Supplement: Supplementary file 4 [file Image1.TIF]
